# Detecting myasthenia gravis as a cause of unclear dysphagia with an endoscopic tensilon test

**DOI:** 10.1177/17562864211035544

**Published:** 2021-08-11

**Authors:** Tobias Warnecke, Sun Im, Bendix Labeit, Olga Zwolinskaya, Sonja Suntrup-Krüger, Stephan Oelenberg, Sigrid Ahring, Matthias Schilling, Sven Meuth, Nico Melzer, Heinz Wiendl, Tobias Ruck, Rainer Dziewas

**Affiliations:** Department of Neurology with Institute of Translational Neurology, University of Muenster, Albert-Schweitzer-Campus 1, Building A1, Münster, 48149, Germany; Department of Rehabilitation Medicine, Bucheon St. Mary’s Hospital, College of Medicine, The Catholic University of Korea, Seoul, Republic of Korea; Department of Neurology with Institute of Translational Neurology, University of Muenster, Muenster, Germany; Institute of Biomagnetism and Biosignal analysis, University of Muenster, Muenster, Germany; Department of Neurology with Institute of Translational Neurology, University of Muenster, Muenster, Germany; Department of Neurology with Institute of Translational Neurology, University of Muenster, Muenster, Germany; Institute of Biomagnetism and Biosignal analysis, University of Muenster, Muenster, Germany; Department of Neurology with Institute of Translational Neurology, University of Muenster, Muenster, Germany; Department of Neurology with Institute of Translational Neurology, University of Muenster, Muenster, Germany; Department of Neurology with Institute of Translational Neurology, University of Muenster, Muenster, Germany; Department of Neurology, Heinrich-Heine University of Duesseldorf, Duesseldorf, Germany; Department of Neurology, Heinrich-Heine University of Duesseldorf, Duesseldorf, Germany; Department of Neurology with Institute of Translational Neurology, University of Muenster, Muenster, Germany; Department of Neurology, Heinrich-Heine University of Duesseldorf, Duesseldorf, Germany; Department of Neurology and Neurorehabilitation, Hospital Osnabrueck, Osnabrueck, Germany

**Keywords:** differential diagnosis, dysphagia, flexible endoscopic evaluation of swallowing, myasthenia gravis, tensilon

## Abstract

**Aims:**

The flexible endoscopic evaluation of swallowing-tensilon test (FTT) was developed to diagnose myasthenia gravis (MG) in patients with unclear pharyngeal dysphagia. The purpose of this study was to determine sensitivity and specificity of the FTT and compare its diagnostic validity with that of other diagnostic markers.

**Methods:**

In this single-centre pragmatic clinical cohort study, a total of 100 patients with unclear pharyngeal dysphagia were eligible to undergo FTT. All patients were subjected to FTT and subsequently followed up clinically. FTT was considered positive if a significant improvement of pharyngeal swallowing function could be objectified endoscopically upon administration of edrophonium chloride. In addition, repetitive nerve stimulation test and serum MG antibody analysis were conducted.

**Results:**

All subjects (mean age 62.5 ± 14.1 years, female 33) underwent FTT without any complications. According to the results of the diagnostic procedures and based on long-term clinical follow-up for at least 3 years, 51 patients were finally diagnosed with MG. The sensitivity and specificity for the FTT was 88.2% and 95.9%, respectively. Application of the Cochran’s *Q* test showed statistically significant heterogeneity among the diagnostic tests, with results indicating FTT performance to be more accurate than the repetitive nerve stimulation results (*p* < 0.001) and comparable with serum antibody tests (*p* > 0.99).

**Conclusion:**

FTT has excellent clinical properties to be used routinely in the assessment of dysphagia with isolated or predominant pharyngeal muscle involvement allowing rapid and accurate diagnosis of MG.

## Introduction

Approximately a quarter of patients with myasthenia gravis (MG) presents with predominant oropharyngeal bulbar weakness and up to 67% develop dysphagia during the course of their disease.^1^ When pharyngeal dysphagia is the sole or predominant symptom, establishing the diagnosis of MG may be difficult,^2^ especially since it occurs in various diseases.^3^ However, a delay in the diagnosis can lead to life threatening myasthenic crisis.^4^ A valid diagnostic approach for this patient group is therefore urgently needed.^5,6^

The ‘classical’ tensilon test – one of the standard diagnostic procedures in MG – assesses whether intravenous administration of edrophonium chloride leads to an improvement in muscle strength.^7^ Unlike the ocular or limb muscles, improvement of pharyngeal hypocontractility after tensilon application is much more sophisticated to evaluate, as the pharyngeal muscles are not directly visible and MG patients often do not perceive their swallowing dysfunction adequately.^8^ Therefore, instrumental assessment such as flexible endoscopic evaluation of swallowing (FEES) or videofluoroscopy is recommended as diagnostic gold standard in the evaluation of myasthenic dysphagia.^9^

We previously described the FEES-tensilon test (FTT) as a novel standardized diagnostic protocol applying edrophonium chloride during a FEES examination with the aim to objectively evaluate improvement of swallowing function.^8^ Recently, it was demonstrated that this protocol is associated with an excellent inter- and intra-rater reliability.^9^ However, the diagnostic accuracy of this test has not yet been investigated. The aim of this study was therefore to validate the FTT in a cohort of patients with unclear pharyngeal dysphagia as leading symptom.

## Methods

### Participants

**Figure 1.**
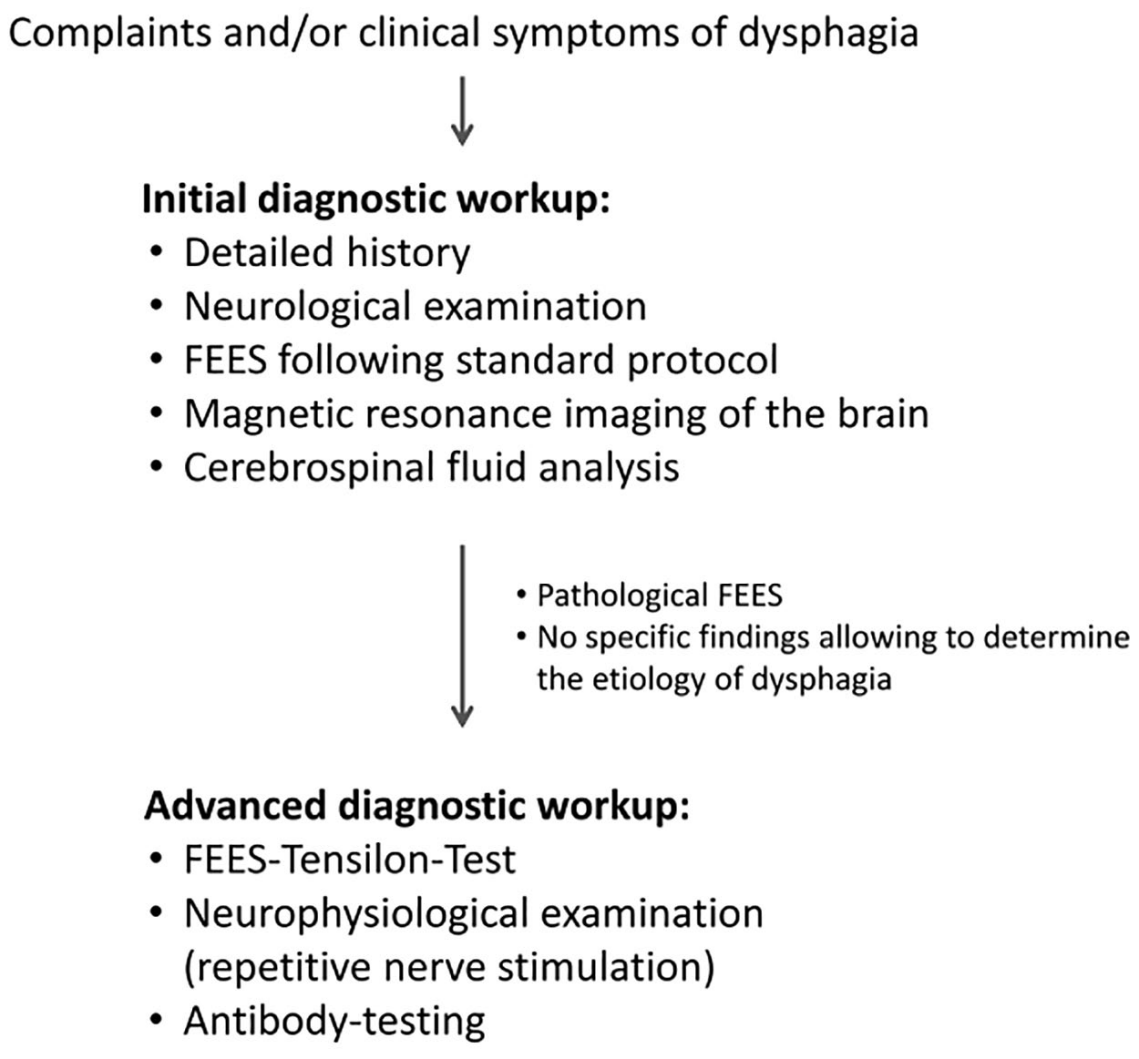
Diagnostic workup of patients with unclear pharyngeal dysphagia.

All examinations were part of our local routine procedure for dysphagia assessment. Patients were examined at University Hospital Muenster between 2010 and 2015 and retrospectively included in the analysis. During this period patients with unclear pharyngeal dysphagia were assessed according to the diagnostic algorithm illustrated in Figure 1. Patients were included only if, after clinical history and detailed neurological examination, the cause of pharyngeal dysphagia remained undefined, whereas patients with pre-existing MG or other known underlying diseases of dysphagia were excluded. If individuals showed clinical features that were suggestive of other disorders for example bradykinesia, rigidity, tremor, ataxia, hemiparesis, cranial nerve palsy, or pyramidal signs, they were excluded from the study. Also, if individuals showed definite ‘classical’ characteristics of MG, such as diplopia, ptosis, or fluctuating limb muscle weakness aggravated by exercise and relieved after periods of rest, they were excluded. To rule out other neurological disorders, patients were excluded if the had severely abnormal findings in cranial magnetic resonance imaging (MRI) or cerebrospinal fluid (CSF) analysis. Of all patients who met these inclusion and exclusion criteria, patients were further excluded if there was incomplete data, either because the FTT was rejected by the patient, was not performed due to clinical risk factors of a cholinergic reaction, or no information was available regarding follow-up examinations for at least 3 years. Otherwise, all patients were included in the analysis.

### Flexible endoscopic evaluation of swallowing

Following a clinical swallowing examination, a baseline FEES examination was performed according to the standard protocol as described by Langmore.^10^ After evaluation of pharyngeal anatomy and physiology, pharyngeal swallowing function was assessed with three different bolus consistencies, i.e. puree, solids and thin liquids. During the FEES exam, saliva management, movement of pharyngeal and laryngeal structures, tongue base retraction and sensation were assessed. Salient findings of dysphagia such as premature spillage, delayed swallowing reflex, penetration/aspiration and residue were rated.^10^ Penetration/aspiration events were further categorised as either being at pre-, intra- or postdeglutitive stages. If residues of boluses were observed after swallowing, the location and amount were documented. A patient was scored as positive on the fatigable swallowing test, if the amount of residue increased over up to 30 consecutive swallows to >50% of bolus size.^11^ Based on the FEES results, the overall severity of dysphagia was assessed using the following ordinal scale: 0, normal: no signs of dysphagia; (1) mild: signs of dysphagia (premature spillage, pharyngeal residue) without penetration or aspiration; (2) moderate: penetration and/or aspiration of one bolus consistency; (3) severe: penetration and/or aspiration of multiple bolus consistencies.

### The FEES-tensilon test

To assess tensilon responsiveness of pharyngeal dysphagia the FTT protocol allowing for a standardised evaluation of swallowing function before and after tensilon administration was performed as described in detail elsewhere.^8^ In brief, the FTT was conducted first by administering a placebo injection of saline. Then after clearance of the hypopharynx, edrophonium chloride, i.e., tensilon, was administered rapidly intravenously to a total cumulative dosage of 10 mg. The FTT was considered ‘positive’, if a clear improvement of dysphagia was observed immediately after injection compared with the result obtained with the placebo injection. For this, the FEES findings of at least one of the following main pathologies had to improve significantly: (1) premature bolus spillage, (2) residue in the piriform sinus or the valleculae according to the Yale Pharyngeal Residue Scale or, in analogy to this scale in the case of residue at the level of the lateral cannels or the epiglottis, (3) penetrations or aspirations.^12^ The full criteria for improvement of swallowing function are defined in detail elsewhere.^8,9^

The FTT was performed and interpreted by a speech language pathologist together with a trained neurologist at the bedside, both with >5 years of experience in the field of neurogenic dysphagia and FEES.

### Final diagnosis and grading of MG

The final diagnosis of MG was established either by a positive laboratory test for specific autoantibodies, i.e. serum anti-acetylcholine receptor (Ach-R) antibodies or serum anti-muscle-specific kinase (MuSK) antibodies. In seronegative patients the diagnosis was established by (1) either a positive decrement response from the neurophysiological assessment [repetitive nerve stimulation (RNS)], (2) or a characteristic response to therapy, i.e. definite improvement following administration of an oral cholinesterase inhibitor (pyridostigmine) or immunosuppressive treatment.^13^ The RNS test was carried out following a standard protocol and a decrement response in the amplitude of more than 10% was considered positive.^14^ The RNS was performed on the accessory and facial nerves and recording was obtained from the trapezius and nasals muscles, respectively, using the Dantec™ Keypoint® G4 EMG/NCS/EP workstation.

The severity of MG was graded according to the modified Myasthenia Gravis Foundation of American (MGFA) classification.^15^ Those patients whose clinical features, laboratory findings, neurophysiological tests, and/or response to therapy were not compatible with a definite diagnosis of MG, underwent further diagnostic workup, focussing, amongst others, on motor neuron disorders, myopathy, hereditary disorders or paraneoplastic syndromes. All patients were followed up for a minimum of 3 years. FTT, neurophysiological examination, and blood collection for antibody testing were performed within a maximum of 5 consecutive days.

### Statistical analysis

All statistical analyses were carried out with SPSS 12.0 for WINDOWS (SPSS Inc., Chicago, IL, USA). Statistical analysis for group comparisons between those diagnosed as MG (+) and MG (−) were performed with the independent *t* test for interval values; Mann–Whitney *U* test for ordinal values and chi square or Fischer’s exact test for nominal values. Sensitivity and specificity levels (95% CI) were analysed using the 2 × 2 test to evaluate the diagnostic parameters. To obtain the overall measures of the diagnostic accuracy and predictive abilities of these diagnostic tests, diagnostic parameters were calculated for the following parameters: the FTT, the serum antibody tests; RNS and the fatigable swallowing test. Comparison of the FTT with the other diagnostic tests was performed to determine whether significant differences existed in the classification results among these tests with the Cochran’s *Q* test.^16^

### Ethics

Retrospective data analysis was approved by the ethics committee of the ‘Ärztekammer Westfalen-Lippe and Westfalian Wilhelm University of Münster’ (AZ: 2016-391-f-S). Due to the retrospective design, the ethics committee waived the need for informed consent.

### Data availability

All relevant data are published in this manuscript.

## Results

### Study cohort and baseline FEES results

**Figure 2.**
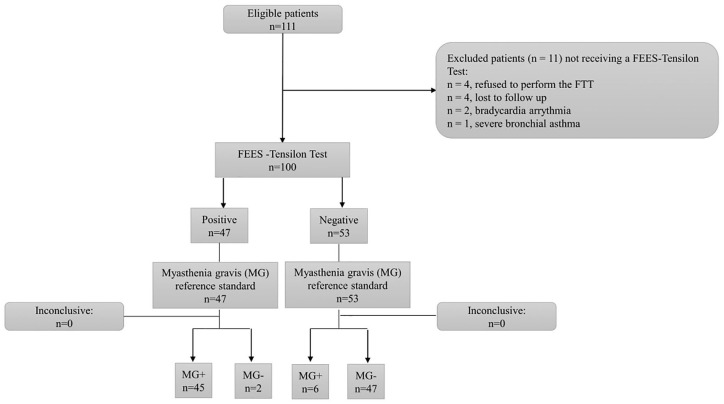
STARD flow diagram. FEES, flexible endoscopic evaluation of swallowing; FTT, flexible endoscopic evaluation of swallowing-tensilon test; MG, myasthenia gravis; STARD, standards for the reporting of diagnostic accuracy.

A total of 100 patients with pharyngeal dysphagia as the predominant or sole symptom underwent FTT. Figure 2 shows the STARD (standards for the reporting of diagnostic accuracy – see Supplemental material for STARD checklist) diagram to report the flow of participants through the study. The further comprehensive diagnostic workup after FTT revealed MG in 51 of these cases. Table 1 summarizes the clinical characteristics of the MG patients. Those who did not meet the diagnostic criteria of MG (*n* = 49) were later diagnosed as follows: 19 motor neuron disorders (amyotrophic lateral sclerosis = 10, Kennedy disease = 4, other motor neuron disorders = 5), 13 myopathies, 6 neuropathies (polyneuropathies or cranial neuritis), 2 multiple system atrophy, 1 limbic encephalitis, 1 Lambert-Eaton myasthenic syndrome, 2 somatoform disorders and 5 unclear diagnosis. Between the MG *versus* non-MG group, no significant differences in mean age (62 ± 15.3 *versus* 63.1 ± 12.9) or gender (male = 70.5% *versus* 63%) were observed. Tables 2 and 3 summarize the findings of the clinical swallowing examination and the baseline FEES. Myasthenic dysphagia involved both the oral phase (as observed by insufficient velopharyngeal closure and premature spillage of the bolus) and the pharyngeal phase (as observed by severe aspiration and residues within varying pharyngeal locations between consecutive swallows not showing a distinct pattern of residue accumulation). The fatigable swallowing test was positive in 38 (74%) among those who were finally diagnosed as having MG.

**Table 1.** Clinical and laboratory characteristics of MG patients. Results are presented as mean or median (IQR), or number (percentage).

MGFA	II_b_^a^	III_b_^b^	IV_b_	V^c^	*p* value
Number (%)	18 (35.3)	12 (23.5)	12 (23.5)	9 (17.7)	
Dysphagia severity	1 (1–2)	2 (1.5–3)	2.5 (1–3)	3 (2–3)	0.008
Thymoma	4 (22.2)	1 (8.3)	5 (41.7)	3 (33.3)	0.289
Positive RNS	9 (50.0)	9 (75.0)	5 (41.7)	3 (33.3)	0.250
Positive serum antibody test	17 (94.4)	12 (100.0)	10 (83.3)	9 (100.0)	0.384
Positive FEES-tensilon test	18 (100)	11 (91.7)	10 (83.3)	6 (66.7)	0.035

a Mild weakness, predominantly affecting oropharyngeal, respiratory muscles or both.

b Moderate weakness, predominantly affecting oropharyngeal, respiratory muscles or both.

c Intubation with or without mechanical ventilation

_b_severe weakness, predominantly affecting oropharyngeal, respiratory muscles or both.

FEES, flexible endoscopic evaluation of swallowing; IQR, interquartile range; MG, myasthenia gravis; MGFA, Myasthenia Gravis Foundation of American; RNS, repetitive nerve stimulation.

**Table 2.** General demographic characteristics of patients referred for dysphagia due to undefined causes. Results are presented as mean (SD), median (IQR) or number (percentage). Group comparisons performed *via* independent *t*-test for interval values; Mann–Whitney test for ordinal values and chi square or Fischer’s exact test for nominal (proportional) values if *n* < 5.

Demographics	MG (*n* = 51)	Non MG (*n* = 49)	*p* value
Clinical characteristics			
Duration in months	7 (4–42)	24 (9.5–60)	0.066
Swallowing complaints	40 (78.4)	30 (61.2)	0.061
Weight loss (kg)	2 (5.1)	1.4 (4.5)	0.050
History of pneumonia	6 (11.8)	3 (6.1)	0.488
Clinical swallowing evaluation			
Mastication fatigue, or paresis at rest	17 (32.3)	1 (2)	<0.001*
Positive aspiration at screening	24 (47.1)	10 (20.4)	0.005*

* *p* values < 0.05.

IQR, interquartile range; MG, myasthenia gravis; SD, standard deviation.

**Table 3.** Dysphagia characteristics from the baseline FEES findings. Results are presented as mean (SD) or median (IQR), or number (percentage). Group comparisons performed *via* independent *t*-test for interval values; Mann-Whitney test for ordinal values and chi square or Fischer’s exact test for nominal (proportional) values if *n* < 5.

Items	MG (*n* = 51)	Non MG (*n* = 49)	*p* value
Dysphagia severity	2 (1–3)	1 (0–2)	<0.001*
Pathological FEES findings			
Physiological assessment			
Saliva aspiration	7 (13.7)	6 (12.2)	0.826
Weak velopharyngeal closure	14 (27.5)	1 (2.0)	<0.001*
Weak pharyngeal contraction	5 (9.8)	0 (0.0)	0.057
Weak base of tongue movement	9 (17.6)	3 (6.1)	0.159
Pharyngeal hypaesthesia	2 (3.9)	0 (0.0)	0.459
Bolus swallowing			
Premature spillage	31 (60.7)	18 (36.7)	0.016*
Delayed swallow reflex	8 (15.7)	5 (10.2)	0.415
Bolus type aspiration			
Solid	5 (9.8)	2 (4.1)	0.437
Semisolid	10 (19.6)	3 (6.1)	0.045
Liquid	16 (31.3)	4 (8.1)	0.004*
Residue after swallowing			
Residue severity grade	2 (1–2)	1 (0–2)	0.009*
Vallecular	14 (27.5)	18 (36.7)	0.057
Pyriform	3 (5.8)	0 (0.0)	0.243
Both	24 (47.0)	13 (26.6)	0.034

* *p* values < 0.05.

FEES, flexible endoscopic evaluation of swallowing; IQR, interquartile range; MG, myasthenia gravis; SD, standard deviation.

### FTT results

Apart from mild and temporary muscarinic side effects such as transient bradycardia, all patients underwent FTT without any relevant complications; none of the patients were antagonized with atropine. Among them, 88.2% of patients from the MG group showed a clear improvement after tensilon administration. In the non-MG group, 95.9% showed no improvement of swallowing after tensilon application.

### Autoantibody testing

Three patients had missing data for the anti-MuSK antibody results, but all patients had serum analysis results available for the Ach-R antibody tests. A total of 47 patients (92.2%) in the MG group were positive for Ach-R antibodies and 4 patients (7.8%) tested positive for anti-MuSK antibodies. In addition, 13 patients (25.5%) tested positive for titin antibodies. Of the 4 patients who showed negative results for the Ach-R antibody, 1 tested positive for MuSK antibodies, resulting in a total of 48 patients being positive for the serum antibody test. Three patients (5.8%) in the MG (+) were seronegative, of whom two showed a positive FTT; the other patient was negative to all tests, but was later diagnosed with MG after showing symptomatic improvement to long-term medication with oral cholinesterase inhibitor.

### RNS results

Three patients had missing data for the RNS studies. In total, 26 patients (50.9%) had a MG-suggestive RNS result; 17 patients (65.4%) showed a positive decrement response of the musculus nasalis, 3 patients (11.5%) of the musculus trapezius and 6 patients (23.1%) of both tested muscles.

### Diagnostic accuracy

FTT provided excellent diagnostic accuracy (Table 4) with a sensitivity of 88.2%, a specificity of 95.9%, a positive predictive value of 95.7% and a negative predictive value of 88.7%. Application of Cochran’s *Q* test showed statistically significant heterogeneity among the diagnostic tests. A *post hoc* McNemar’s test showed that there were significant differences between the FTT *versus* the RNS diagnostic parameters (*p* < 0.001). However, the FTT showed results comparable with those of the serum antibody tests (*p* > 0.999) and the clinical fatigable swallowing test (*p* = 0.450).

**Table 4.** Diagnostic parameters (95% CI) of the FTT, serum antibody, RNS and fatigable swallowing test.

Diagnostic test		MG (+)	MG (−)	Sensitivity	Specificity	PPV	NPV
FTT	(+)	45	2	0.882	0.959	0.957	0.887
	(−)	6	47	(0.761–0.956)	(0.860–0.995)	(0.855–0.995)	(0.770–0.957)
Serum antibody test	(+)	48	0	0.941	1.000	1.000	0.942
	(−)	3	49	(0.8381–0.988)	(1.000–1.000)	(0.926–1.000)	(0.841–0.988)
RNS^a^	(+)	26	0	0.510	1.000	1.000	0.662
	(−)	25	49	(0.366–0.653)	(1.000–1.000)	(1.000–1.000)	(0.543–0.768)
Fatigable swallow	(+)	38	13	0.745	0.735	0.745	0.735
	(−)	13	36	(0.604–0.857)	(0.589–0.851)	(0.604–0.857)	(0.589–0.851)

a Statistically different from the FTT, *p* < 0.001 (Cochran’s Q test, *post hoc* McNemar’s test).

CI, confidence interval; FEES, flexible endoscopic evaluation of swallowing; FTT, FEES-tensilon test; MG, myasthenia gravis; NVP, negative predictive value; PPV, positive predictive value; RNS, repetitive nerve stimulation.

## Discussion

The results of this study indicate that the FTT is a useful tool to diagnose MG in patients with unclear dysphagia. It exhibits excellent diagnostic accuracy with a sensitivity of 88.2% and a specificity of 95.9%. The test procedure was safely applied to MG patients across various stages from MGFA class II to V with no clinically relevant complications. Our findings therefore confirm this test to be a valid, and technically easy tool that offers immediate bedside results. The comparison of these tests showed that the diagnostic accuracy of the FTT in our study population was comparable with that of antibody testing and superior to RNS results.^17^

The diagnostic parameters of the FTT in this study were found to be like those reported for the standard tensilon test with sensitivity levels of 88% and specificity levels of 97%.^18^ Besides in the diagnosis of generalized MG, past studies have shown that tensilon testing can be useful for the diagnosis of ocular MG,^7^ but our study is the first to report diagnostic validity in the patient group, with predominant bulbar symptoms resulting in pharyngeal dysphagia. Since pharyngeal swallowing is not visible from the outside, the standard tensilon-test has inherent limitations to record improvement of bulbar muscle function without the use of an instrumental swallowing test. In this context, the FTT provides an objective and reliable visualization of swallowing improvement.

Many guidelines recommend the use of RNS decrement or the Ach-R antibody tests as diagnostic gold standard in MG.^2,19–21^ However, these test procedures have important limitations in the group of patients with oropharyngeal dysphagia as main clinical symptom: the decrement response in the RNS may vary according to the muscles tested.^22^ Although the nasalis muscle is recommended for patients with bulbar disease, the sensitivity is reported to be as low as 46%.^17^ This is in line with RNS results from our study, which also indicate a low sensitivity. However, sensitivity may increase if RNS is performed in the muscle groups that are clinically affected. In cases of bulbar MG hypoglossal RNS was reported to correlate with bulbar dysfunction.^23^ However, performing direct RNS in the bulbar muscles is technically challenging, prone to artefacts and may cause discomfort to patients. In contrast, the FTT is technically less demanding than RNS and causes minimal discomfort to the patient. As shown in our study, no patient dropout was observed during the FEES-tensilon procedure.

Along with RNS, the detection of serum antibodies is a standard diagnostic test in MG.^2,19–21^ MG antibodies are detected in approximately 88% of patients with clinical features of MG.^24^ Due to the high positive predictive value, the detection of antibodies essentially confirms the diagnosis of MG and obviates the need for further testing.^2^ However, the negative predictive value in cases without detectable antibodies is less conclusive and up to 15% of MG patients are seronegative.^24^ Also, patients can be falsely classified as seronegative due to immunosuppression or if the test is done too early in the course of the disease.^2^ Another disadvantage of antibody testing in the clinical routine is the long time required to wait for the final serum results, which sometimes may take several weeks. However, this time interval can be a crucial setback, especially for patients under critical medical condition such as severe dysphagia with saliva aspiration that may subsequently require intubation. In contrast, the FTT provides immediate results also at an early disease stage and can facilitate urgent therapy decisions while other diagnostics are pending.

In addition to the validation of the FTT, this study provides a detailed description of pharyngeal dysphagia phenotypes from the largest cohort of endoscopically assessed MG patients. Oropharyngeal dysphagia was present across various MG disease severities and resulted in similar subjective complaints and clinical presentation compared with patients who were later diagnosed with motor neuron disorders or myopathies. This is consistent with reports of dysphagia as an initial or sole symptom in these patient groups.^25–27^ Nevertheless, the MG patients presented endoscopically significantly more often with a weak velopharyngeal closure and with a more severe grade of dysphagia during baseline FEES. Like muscle fatigue described in peripheral muscles, pharyngeal muscle fatigue after multiple swallows was detected frequently with the standardized fatigable swallowing test, and resulted in the build-up of residues in the valleculae and pyriform sinus. However, our results revealed only moderate sensitivity and specificity for the diagnosis of MG in our study cohort, indicating that the fatigable swallowing test may give important diagnostic hints but is insufficient as a stand-alone diagnostic in bulbar MG patients. Further, sensitivity of the FTT for diagnosis of isolated bulbopharyngeal MG decreased with increasing severity of dysphagia. One possible explanation for this finding is that the patients with severe dysphagia often had a global dysphagia pattern with several and heterogenous pathologies. In addition, prolonged dysphagia with associated intubation may have lead to further secondary dysphagia components such as intubation-related damage to the mucosa. This may have made it more difficult to identify and improve individual dysphagia pathologies in the FTT as the severity of dysphagia increased. Another possible explanation could be that severe courses of MG with associated severe dysphagia is more often also associated with systemic disease involvement, so that, in severe isolated dysphagia cases, patients with MG as the cause of dysphagia were less frequent. In contrast to FTT, the sensitivity for antibody testing remained consistently high with increasing disease severity. This could imply that, especially in severe disease stages, the sensitivity of antibody testing is superior to FTT.

Some limiting factors need to be considered in this study. Only MG patients with predominant bulbar manifestations were included. The diagnostic parameters of the applied tests may differ from those with more prominent ocular or generalized forms of MG. In the US, edrophonium chloride was terminated by the Food and Drug Administration in view of false-positive test results and due to other available diagnostic gold standards such as antibody detection and is currently not commercially available.^28^ Nevertheless, in our study the FTT showed good diagnostic ratios. However, it must be taken into account that all examiners had several years of experience with FEES diagnostics, and that the diagnostic ratios of less experienced examiners may deviate considerably. Besides RNS, single fibre electromyography is the most sensitive electrodiagnostic method,^29^ but it requires specialized equipment and training and was not performed in this study.

## Conclusion

In summary, the results of this study confirm that the FTT is a feasible and valid diagnostic tool to detect MG in patients with unclear dysphagia. It shows diagnostic accuracy comparable with that of serum antibody tests and should therefore be considered as adequate diagnostic procedure in patients with predominant bulbar MG.

## Supplemental Material

sj-pdf-1-tan-10.1177_17562864211035544 – Supplemental material for Detecting myasthenia gravis as a cause of unclear dysphagia with an endoscopic tensilon test Click here for additional data file. Supplemental material, sj-pdf-1-tan-10.1177_17562864211035544 for Detecting myasthenia gravis as a cause of unclear dysphagia with an endoscopic tensilon test by Tobias Warnecke, Sun Im, Bendix Labeit, Olga Zwolinskaya, Sonja Suntrup-Kröger, Stephan Oelenberg, Sigrid Ahring, Matthias Schilling, Sven Meuth, Nico Melzer, Heinz Wiendl, Tobias Ruck and Rainer Dziewas in Therapeutic Advances in Neurological Disorders
